# Impacts of Traditional Warm-Up and Post-Activation Potentiation on Muscle Endurance During the Back Squat: Response of Blood Lactate, Perceived Effort, and Time Under Tension

**DOI:** 10.3390/jfmk10020188

**Published:** 2025-05-24

**Authors:** Taianda M. Amorim, Alexandre V. Gurgel, Viviane Faleiro, Thiago T. Guimarães, Estêvão R. Monteiro, Felipe G. Teixeira, Bruno Jotta, Tiago C. Figueiredo, Raquel C. Castiglione, Silvio R. Marques-Neto

**Affiliations:** 1 Programa de Pós-graduação em Ciências da Atividade Física, Universidade Salgado de Oliveira (UNIVERSO), Niterói 24030-060, Brazil; taiandapersonal@hotmail.com (T.M.A.); coachgurgel@gmail.com (A.V.G.); vivivolei87@hotmail.com (V.F.); thiago.guimaraes@nt.universo.edu.br (T.T.G.); 2 Laboratório de Fisiologia do Exercício (LAFIEX), Universidade Estácio de Sá, Rio de Janeiro 20771-004, Brazil; fegute1@hotmail.com (F.G.T.); brunojottac@mail.com (B.J.); tc-figueiredo@uol.com.br (T.C.F.); 3 Postgraduate Program in Rehabilitation Science (PPGCR/UNISUAM), Centro Universitário Augusto Motta, Rio de Janeiro 21041-020, Brazil; profestevaomonteiro@gmail.com; 4 Laboratório de Pesquisas Clínicas e Experimentais em Biologia Vascular (BioVasc), Universidade do Estado do Rio de Janeiro (UERJ), Rio de Janeiro 20550-013, Brazil; rccastiglione@gmail.com

**Keywords:** post-activation potentiation, warm-up strategies, muscular endurance, back squat, lactate, perceived exertion, strength training, time under tension

## Abstract

Background: Warm-up strategies are essential for optimizing strength-training performance. Traditional warm-ups improve neuromuscular readiness, whereas post-activation potentiation (PAP) has been proposed to acutely enhance muscular output. This randomized crossover study compared the acute effects of traditional and PAP-based warm-ups on local muscular endurance (LME) during free weight back squats in resistance-trained men. Methods: Twelve trained males (age: 41.3 ± 5.7 years; one repetition maximum squat: 129.3 ± 14.3 kg) completed three randomized squat sessions: mobility with LME (M + LME), traditional warm-up with LME (T + LME), and PAP with LME (PAP + LME). The sessions were spaced 48 h apart. Outcomes included the number of repetitions, blood lactate concentration, time under tension (TUT), perceived exertion through OMNI Resistance Exercise Scale (OMNI-RES), and pain perception through visual analogue scale (VAS). One-way ANOVA and partial eta-squared (η^2^p) were used for statistical analyses. Results: PAP + LME significantly increased the number of repetitions (15.63 ± 3.66) compared to both M + LME (12.38 ± 3.89) and T + LME (13.63 ± 3.82; *p* < 0.0001). Blood lactate levels were significantly higher in PAP + LME (8.98 ± 3.87 mmol/L) compared to M + LME (5.08 ± 0.97 mmol/L; *p* = 0.01). TUT was significantly shorter in both the PAP + LME and T + LME groups than in the M + LME group (*p* < 0.05). VAS scores were higher after PAP + LME (8.50 ± 0.45) than after M + LME (6.50 ± 1.20; *p* = 0.02), while OMNI-RES scores did not differ significantly between the protocols. Conclusions: Both traditional and PAP-based warm-ups improved squat LME compared with mobility alone. PAP elicited greater repetition performance and metabolic stress but also increased discomfort. Warm-up selection should align with training goals, balancing performance benefits and perceived fatigue.

## 1. Introduction

Strength training (ST) is a widely practiced modality that promotes both central (neural) and peripheral (skeletal muscle) adaptations, resulting in increased neuromuscular strength, muscle hypertrophy, and improved body composition [1]. Beyond musculoskeletal benefits, ST is also associated with increased bone mineral density, enhanced metabolic rate, and improved psychological well-being, making it essential for both athletes and the general population [2].

Among ST exercises, squats are among the most functionally relevant and frequently applied movements across fitness, rehabilitation, and sports performance settings [3]. However, to maximize their effectiveness, training variables such as execution technique, load intensity, movement velocity, range of motion, and warm-up strategies must be precisely manipulated [4].

Warm-up is a critical preparatory strategy that enhances physical performance and reduces injury risk. Physiologically, it elevates muscle temperature, improves elasticity, enhances metabolic efficiency, increases oxygen delivery, and primes the neuromuscular system for motor unit recruitment [5,6]. Common warm-up modalities in ST include aerobic exercise, static stretching (SS), dynamic stretching (DS), specific warm-up drills, and joint mobility routines [5]. Despite their widespread use, the relative effectiveness of these methods in optimizing neuromuscular activation and performance is still under debate [7].

More recently, post-activation potentiation (PAP) has emerged as an alternative warm-up strategy aimed at acutely enhancing muscle performance. PAP is defined as a transient increase in muscle force production following a high-intensity conditioning stimulus [6]. Although historically associated with explosive activities (e.g., sprints, Olympic lifts), PAP has gained attention in submaximal strength exercises such as squats [8].

The physiological mechanisms underlying PAP include increased muscle temperature, enhanced neuromuscular activation, and improved responsiveness of contractile proteins [9]. These effects collectively contribute to greater force output and improved muscular performance. While the physiological mechanisms of PAP are traditionally linked to improvements in power and explosive performance, recent studies have suggested that PAP can also enhance muscular endurance by increasing the initial motor unit recruitment and optimizing neuromuscular efficiency, thereby delaying fatigue onset during submaximal efforts. For instance, Boullosa et al. [10] demonstrated that PAP-induced neural adaptations may contribute to improved endurance performance by enhancing the rate coding and synchronization of motor units, particularly in trained individuals.

Despite its potential, the application of PAP in strength training remains controversial due to inconsistent findings across studies. Variables such as intensity of the conditioning stimulus, rest interval duration, biomechanical specificity, and individual training status modulate the effectiveness of PAP [11]. Notably, insufficient recovery may lead to fatigue, counteracting the potentiating effects.

Traditional warm-ups aim to progressively prepare the body for physical effort, while PAP protocols seek to enhance acute neuromuscular performance. Although both approaches are valid, determining which is superior for muscular endurance and strength performance remains unresolved [12]. Some evidence suggests that combining general and specific warm-up components may optimize readiness while minimizing fatigue [13].

The vector theory of specificity posits that warm-up movements should replicate the target task to maximize effectiveness [14]. This supports the use of movement-specific warm-ups, particularly in squat-based exercises. However, the current literature lacks consensus on whether PAP or traditional warm-up methods offer superior outcomes in endurance-oriented ST.

Given the ongoing debate and methodological variability in the literature, it is crucial to compare the acute effects of traditional warm-up methods and PAP protocols in a controlled ST context. Therefore, the main aim of this study was to investigate the acute effects of different warm-up strategies—including both traditional approaches and PAP—on muscular performance during squat exercises.

We hypothesized that the post-activation potentiation (PAP) warm-up protocol would be more effective than traditional warm-up methods in enhancing muscle endurance performance during the back squat exercise. To test these hypotheses, we adopted a within-subject crossover design in which trained male participants underwent three squat sessions preceded by distinct warm-up protocols. By comparing objective (TUT, repetitions, lactate) and subjective (OMNI-RES, VAS) markers across conditions, our methodology was tailored to examine whether PAP elicits superior acute performance outcomes compared to traditional and mobility-based warm-ups.

Specifically, we expected that the PAP condition would result in increased time under tension (TUT), a higher number of repetitions, and elevated blood lactate concentrations, indicating greater metabolic demand. Additionally, we anticipated that perceived effort—assessed via OMNI-RES and VAS scales—would reflect the increased neuromuscular engagement induced by PAP, supporting its role as a superior warm-up strategy for improving muscular endurance outcomes.

## 2. Methods

### 2.1. Participants

Twelve male individuals participated in this study (Table 1).

This study followed a randomized crossover design, which allowed each participant to serve as their own control and helped reduce interindividual variability. Participants were recruited by convenience sampling; all of them had over three years of consistent resistance training experience. Additionally, they demonstrated a consolidated technique in the back squat and its main variations, minimizing any potential learning effects. In addition, they periodically performed 1 RM tests to adjust their training loads, a common procedure among trained individuals seeking to optimize their performance in competitions. All steps of the tests were explained prior to the start of data collection. The tests were conducted at 48 h intervals, and all tests were performed by the same evaluator at the same time, between 7:00 and 8:00 in the morning. All the participants were part of three research groups, namely local muscular endurance during mobility (M + LME), traditional warm-up (T + LME), and post-activation potentiation (PAP + LME).

The participants were randomly assigned to the order of the three experimental conditions (M + LME, T + LME, and PAP + LME) using a simple randomization process conducted through a computerized random number generator (Excel RAND function). This ensured that the sequence of interventions was counterbalanced across the participants to minimize potential order effects. The sessions were separated by 48 h to allow adequate recovery and reduce carryover effects between the protocols.

The inclusion criteria were as follows: (a) age between 18 and 45 years; (b) practicing RT for at least 3 years; (c) having a weekly frequency of at least three days a week; and (d) the researcher applied the Physical Activity Readiness Questionnaire (PAR-Q), in which the participants had to answer all questions negatively. The participants were asked to complete the PAR-Q test to obtain information related to medication use, cardiovascular risk, and history of injuries.

The exclusion criteria were as follows: (a) osteomioarticular injuries or limitations that could compromise the execution of movements and (b) use of performance-enhancing substances. All the research participants were informed through a written report about the risks and benefits of the study and were asked not to engage in physical activity during the collection period, which could interfere with the results of the study.

Although the participants were instructed to maintain their usual routines, we did not control for dietary intake or sleep habits, which may have influenced physiological responses. Additionally, the short-term nature of the intervention limits the extrapolation of findings to long-term training adaptations.

Besides the PAR-Q, all the participants were instructed to complete an informed consent form (ICF). The project was submitted to the UNIVERSO Research Ethics Committee (CEP/UNIVERSO), and complied with the standards established by Resolution 466/2012, which defines the procedures for research involving human beings. All the subjects signed an informed consent form, with approval from the National Research Ethics Commission—CONEP (No. 64038022.9.0000.5289).

### 2.2. Training and Warm-Up Procedures

On day 1, anthropometric data (height and weight) and personal information (name, age, and strength training experience) were collected. The 1 RM test was performed following the protocol described by Brown and Weir [15]. The participants warmed up with 5 min of treadmill exercise, followed by eight repetitions at 50% of the perceived 1 RM and three repetitions at 70% of the perceived 1 RM. After a 5 min rest, the 1 RM test was conducted by progressively increasing the load in increments of 0.4–5 kg, with a maximum of three to five attempts. The largest successfully lifted weight before concentric failure in the second repetition was recorded as the 1 RM value. To ensure consistency across sessions, the 1 RM was not re-tested but was confirmed verbally with the participant before each session, and no changes in training status were reported during the experimental period.

On day 2, 48 h after the 1 RM test, the participants performed the M + LME protocol. This session began with four mobility exercises (15 repetitions each): frontal and lateral hip mobility, ankle mobility, and suspended hip squat mobility.

During the fourth meeting, 48 h after the M + LME protocol, the participants performed the exercise protocol with a traditional warm-up followed by LME (T + LME), and post-activation potentiation followed by LME (PAP + LME). The T + LME and PAP + LME were randomly performed. During the T + LME session, the participants first performed the same mobility exercises described in the M + LME protocol. They then completed two sets of five back squats at 60% of their 1 RM as part of the traditional warm-up. Immediately after, the load was increased to 75% of 1 RM for the execution of the localized muscle endurance (LME) protocol, which consisted of a single set of back squats performed to concentric failure, without rest.

In the PAP + LME condition, the participants also began with the mobility exercises, followed by two sets of three back squats at 90% of their 1 RM. Immediately after the second set, the load was reduced to 75% of the 1 RM, and the participants performed the LME protocol, again consisting of a single set of back squats to concentric failure, without any recovery interval.

A vertical marker on the squat rack ensured that each subject reached 90° knee flexion. Only full repetitions, defined as knee flexion to 90° (eccentric phase) and full knee extension (concentric phase), were considered valid [16].

During blood collection for lactate analysis, the participants rated perceived pain using the visual analog scale (VAS) and perceived exertion using the OMNI Resistance Exercise Scale.

### 2.3. Blood Lactate Collection

Immediately after squatting to failure, the participants sat on a chair for blood sampling. Capillary blood was collected from the left index finger using an ACCUTREND^®^ Plus device (Indianapolis, IN, EUA). A single blood drop was placed on a BM-LACTATE test strip. The device provided lactate concentration (mM) within one minute. The lancets were discarded after each test.

### 2.4. OMNI-RES Scale and Visual Analog Scale (VAS)

OMNI-RES is an illustrated perceived exertion scale for resistance training. It allows participants to associate subjective effort levels with exercise intensity [17].

The VAS consists of a 10 cm horizontal or vertical line with endpoints labeled “no pain” (0) and “maximum pain” (10) [18].

While blood lactate was being measured, the participants were shown an A4-sized OMNI-RES chart and a VAS chart.

The participants were instructed to indicate their perceived exertion level through the OMNI-RES chart, providing an immediate subjective evaluation of exercise intensity. Their pain level after each session was indicated on the VAS chart, and the researcher measured the distance from the starting point.

### 2.5. Time Under Tension (TUT) Analysis

To assess time under tension (TUT), two-dimensional video analysis was conducted using a Canon ELPH 500 HS digital camera (Melville, NY, USA) with a sampling rate of 120 Hz. The camera was positioned five meters in front of the participant and one meter above the ground. Video recordings were used to capture the full duration of the back squat exercise.

TUT was defined as the total time (in seconds) the muscle remained under tension during the eccentric and concentric phases of movement, from the beginning of the first repetition to the completion of the last repetition within the LME set. Analysis was conducted frame-by-frame using free motion analysis software (e.g., Kinovea, version 2023.1.2), with time stamps extracted for each repetition. The temporal data were summed to yield the total TUT per participant [19,20]. 

To ensure inter-rater reliability in the video analysis of time under tension (TUT), two independent evaluators with prior experience in motion analysis were trained using the same criteria and video examples before data collection. Both evaluators independently analyzed all video recordings using frame-by-frame analysis. Inter-rater reliability was assessed using the intraclass correlation coefficient (ICC), which yielded excellent agreement (ICC = 0.96). In cases of discrepancy greater than 5% between raters, a consensus meeting was held to review the footage and agree on the final value.

### 2.6. Statistical Analysis

All statistical analyses were performed using GraphPad Prism 8 software (version 8.4.2). Descriptive statistics are presented as the means ± standard deviations (SD). Data normality was verified using the Shapiro–Wilk test. For the analysis of continuous quantitative variables (repetitions, lactate, and time under tension—TUT), one-way ANOVA with Tukey’s post-hoc test was used when data presented a normal distribution. For discrete quantitative variables related to subjective perception of effort (OMNI-RES and VAS), which do not assume normality, comparisons were performed using the non-parametric Mann–Whitney test.

The effect size between the groups was established by calculating η^2^p, admitting the following classifications: up to 0.01, small effect size; up to 0.06, medium effect size; and, from 0.14, large effect size. The level of statistical significance adopted for all analyses was *p* < 0.05.

## 3. Results

### 3.1. Comparison of the Effect of Warm-Up Protocols on Squat Muscular Endurance

#### 3.1.1. Number of Repetitions

**Figure 1:**
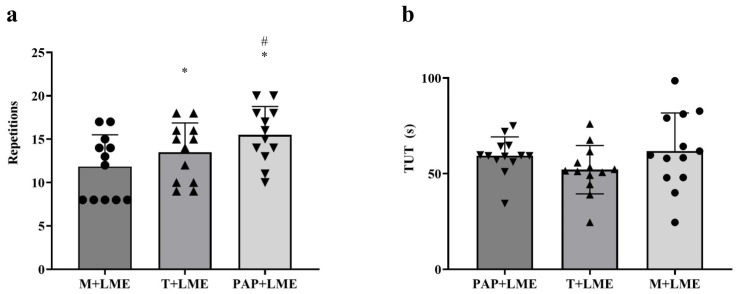
Comparison between mobility only (M + LME), mobility and traditional warm-up (T + LME), and mobility and warm-up with post-activation potentiation (PAP + LME). (**a**) Number of repetitions; (**b**) means of TUT. Comparisons of the means were performed using a one-way analysis of variance. Note: * *p* < 0.0001 vs. M + LME, ^#^ *p* < 0.001 vs. T + LME.

To compare the effects of mobility (M + LME), traditional warm-up (T + LME), and post-activation potentiation (PAP + LME) on squat muscular endurance, the number of repetitions performed in each condition was quantified. Figure 1a illustrates the comparison between the means of the total number of repetitions in the experimental groups. It is observed that both T + LME (13.63 ± 3.82 repetitions) and PAP + LME (15.63 ± 3.66 repetitions) sessions presented a significantly higher number of repetitions compared to M + LME (12.38 ± 3.89; *p* < 0.0001). Additionally, PAP + LME had a greater effect on the number of repetitions than T + LME (*p* < 0.0001).

#### 3.1.2. Time Under Tension

Figure 1b shows a comparison between the TUT means in different training sessions. It is observed that both T + LME (53.25 ± 16.21 s) and PAP + LME (59.79 ± 12.84 s) were not significantly different compared to M + LME (64.23 ± 25.30 s). Additionally, PAP + LME and T + LME did not present significant differences (*p* = 0.94).

### 3.2. Effects of the Warm-Up Protocols on Blood Lactate and Rate of Perceived Exertion

#### 3.2.1. Blood Lactate Levels

**Figure 2:**
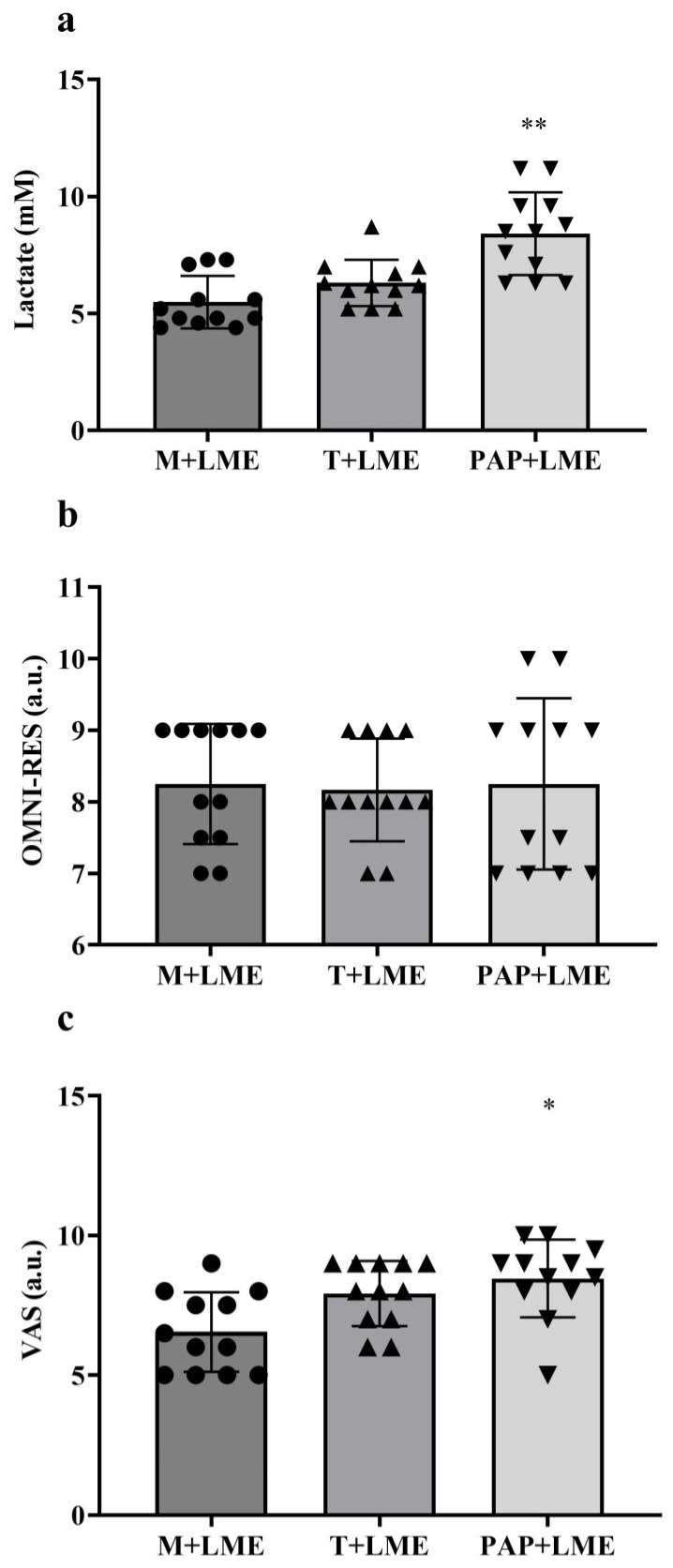
Comparison of acute physiological and perceptual responses between mobility only (M + LME), mobility and traditional warm-up (T + LME), and mobility and warm-up with post-activation potentiation (PAP + LME). (**a**) Blood lactate concentrations were significantly higher in the PAP + LME condition, indicating greater metabolic demand; (**b**) perceived exertion assessed via the OMNI-RES showed no statistically significant differences between the protocols; (**c**) pain perception measured using a VAS scale was significantly higher in the PAP + LME condition compared to M + LME. One-way ANOVA with Tukey’s post-hoc test was used for statistical analysis. Note: * *p* < 0.01 vs. M + LME. ** *p* < 0.001 vs. M + LME.

Considering that blood lactate levels can be influenced by muscular endurance training, Figure 2a shows that the traditional warm-up (T + LME) did not show statistical significance compared to the training session with mobility only (M + LME) (7.31 ± 1.37 mM vs. 5.08 ± 0.97 mM; *p* = 0.15). The training session with warm-up based on the PAP + LME method showed a significant increase in blood lactate concentrations compared to M + LME (8.98 ± 3.87 mM vs. 5.08 ± 0.97 mM; *p* = 0.01). Additionally, the warm-up training session based on the PAP + LME method did not show a significant difference in blood lactate concentrations (8.98 ± 3.87 mM vs. 7.31 ± 1.37 mM; *p* = 0.33) compared to the T + LME training session.

#### 3.2.2. OMNI Resistance Exercise Scale (OMNI-RES)

To analyze the influence of mobility, traditional warm-up, and post-activation potentiation prior to muscular resistance training on the subjective perception of effort using the OMNI-RES, Figure 2b shows the comparison between the means of this variable in the different training sessions. Based on those, the results of the present study showed that regardless of the method adopted prior to the training sessions, there were no significant differences between the means of the OMNI-RES between these groups (M + LME: 8.06 ± 0.31 a.u.; T + LME: 8.00 ± 0.26 a.u.; and PAP + LME: 8.00 ± 0.41 a.u.; *p* = 0.93).

#### 3.2.3. Visual Analog Scale (VAS)

Figure 2c shows a comparison of the means of the VAS after the training sessions of the present study. It is noted that T + LME did not present a significant difference compared to the M + LME training session (8.00 ± 0.42 a.u. vs. 6.50 ± 1.20 a.u.; *p* = 0.07). Regarding the PAP + LME training session, a significant increase in VAS was observed compared to M + LME (8.50 ± 0.45 a.u. vs. 6.50 ± 1.20 a.u.; *p* = 0.02). Additionally, the training session with PAP + LME did not present a significant difference in the VAS compared to T + LME (8.50 ± 0.45 a.u. vs. 8.00 ± 0.42 a.u.; *p* = 0.31).

### 3.3. Effect Size Results

Table 2 shows the effect size calculated using η^2^p. In this sense, the number of repetitions presented a medium effect size for T + LME and PAP + LME compared to M + LME.

Regarding lactate, only the PAP + LME session showed a significant result compared to M + LME, with a medium effect size. In line with the study’s primary objective—to evaluate the acute effects of different warm-up strategies on muscular endurance performance—the results revealed that the PAP + LME condition led to a significantly lower pain perception compared to the M + LME session, as measured using a VAS scale, with a large effect size. Although no statistically significant differences were observed in perceived exertion (OMNI-RES) between the groups, all the conditions demonstrated similarly low scores, with a negligible effect size, suggesting that none of the warm-up strategies imposed excessive subjective fatigue. Moreover, while time under tension (TUT) did not differ statistically between the protocols, the M + LME session showed a noteworthy advantage in performance, indicated by a large effect size when compared to both the T + LME and PAP + LME sessions.

## 4. Discussion

The aim of this study was to investigate the effects of different warm-up protocols, specifically traditional warm-up (T + LME) and post-activation potentiation-based warm-up (PAP + LME) compared with resistance training mobility (M + LME), on local muscular endurance after squat performance. The working hypothesis postulated was that the PAP warm-up protocol could modulate both objective (e.g., muscular endurance and metabolic response) and subjective indicators (perception of exertion and pain), better preparing muscles for subsequent effort. The interpretation of the results, considering the existing literature, allows us to discuss the implications of the findings and suggest directions for future research.

Our results revealed that the PAP + LME condition led to a significantly lower pain perception compared to the M + LME session, as measured using a VAS scale, with a large effect size (η^2^p = 0.35). Although no statistically significant differences were observed in perceived exertion (OMNI-RES) between the groups, all the conditions demonstrated similarly low scores, with a negligible effect size (η^2^p = 0.00), suggesting that none of the warm-up strategies imposed excessive subjective fatigue. Moreover, while time under tension (TUT) did not differ statistically between the protocols, the M + LME session showed a noteworthy advantage in performance, indicated by a large effect size when compared to both the T + LME and PAP + LME sessions (η^2^p = 0.27). These findings suggest that, despite the lack of statistical significance in some variables, warm-up strategies can elicit meaningful practical differences in muscle endurance-related outcomes.

The number of repetitions performed until concentric failure is an important indicator of LME. In this study, both the T + LME and PAP + LME protocols resulted in a higher number of repetitions compared to M + LME. These findings corroborate those of Wilson [19], which indicated that specific and well-structured warm-ups can better prepare muscles for prolonged efforts, thus increasing the ability to perform more repetitions. In addition, McCrary et al. [20] suggested that warm-ups that mimic the main exercise can improve neuromuscular efficiency and coordination, resulting in more effective execution of movement and, consequently, a greater number of repetitions.

The literature suggests that PAP can be beneficial for increasing explosive strength performance, but its impact on long-term muscular endurance needs to be further investigated [10]. Tillin and Bishop [21] suggested that PAP may lead to a decrease in the number of repetitions owing to increased initial fatigue, which was not observed in our study. This may be explained by the intense nature of PAP, which can not only temporarily increase muscle strength and power, but also induce fatigue if not carefully adjusted [21]. Divergently, we found an increase in the number of repetitions with PAP + LME in relation to T + LME, suggesting that when well-adjusted, this technique can improve both muscular strength and muscle endurance.

Although the PAP + LME condition resulted in a statistically significant increase in the number of repetitions compared to the T + LME protocol (15.63 vs. 13.63), the practical relevance of this difference may be limited. An increase of approximately two repetitions, while indicative of a true effect, may not translate into a meaningful improvement in functional capacity or training outcomes, particularly in non-athletic or recreational populations. In elite or high-performance contexts, such a gain might justify the additional complexity and time required to implement post-activation potentiation strategies. However, for general training settings, the logistical demands of PAP protocols may outweigh their marginal benefits. Therefore, the clinical and practical application of PAP should be carefully evaluated based on individual goals, training environments, and available resources.

We could also observe that there was a large effect size of TUT between the M + LME and PAP + LME and between the M + LME and T + LME protocols, with an increased TUT in M + LME, despite no statistical differences. Since TUT is a crucial indicator of strength training efficiency, reflecting the duration during which the muscles are active and supporting load, we can suggest that mobility probably leads to a rapid concentric failure and, therefore, to a lower number of free-weight back squats. To address this concern, we acknowledge that in the absence of statistical significance, large effect sizes must be interpreted cautiously. Although the partial eta-squared value for TUT was large (η^2^p = 0.27), this result was not statistically significant and therefore should not be considered confirmatory evidence of a difference between groups. Instead, we highlight this effect size as a potential trend that warrants further investigation with larger sample sizes. This adjustment ensures statistical rigor while maintaining the relevance of effect size measures as supplementary descriptors of practical magnitude. Traditional warm-up and PAP not only increase muscle resistance in relation to M + LME, but might also be related to a diminished incidence of concentric failure, meaning that the subject is able to perform a higher number of free-weight back squats with a reduced time under tension. In this matter, PAP seems to be more efficient than traditional warm-up.

The literature suggests that increasing TUT can promote greater metabolic stress and microdamage in muscle fibers, resulting in positive adaptations in muscular endurance and hypertrophy [20]. Furthermore, adequate warm-up can improve neuromuscular coordination and movement efficiency, resulting in more economical and effective performance [20]. However, most studies that evaluate TUT effects on muscle hypertrophy and strength increase are designed with equalized training sessions. In our study, training volume was not equalized; that is, our aim was to evaluate whether different types of warm-ups would have an impact on repetition number. Therefore, our results show that muscle metabolic stress was proportional to training volume (number of repetitions × load) instead of being proportional to TUT.

The results of our study are in line with the findings of Schoenfeld et al. [22], who demonstrated that a longer TUT increases metabolic stress, promoting beneficial adaptations such as muscle hypertrophy. This connection between TUT and hypertrophy is also supported by studies indicating that increasing the duration during which muscles are under load can significantly improve muscular endurance [22]. While Schoenfeld et al. [22] support the link between TUT and metabolic stress, it is important to note that their study did not control for the total training volume, which limits the extent to which their results can be directly compared to volume-equated protocols such as ours.

However, several studies have reported conflicting results. For example, Wilk and Zajac [23] suggested that while TUT is important, load intensity and the total work volume are also critical factors for optimizing gains in muscular strength and endurance. They found that varying the combination of TUT, load, and volume could lead to different muscular adaptations, indicating that there is no single optimal method applicable to all individuals. Although the M + LME condition resulted in fewer repetitions compared to the other protocols, no statistically significant difference was observed in TUT. This can be explained by the slower repetition tempo and greater muscle engagement throughout each movement, which likely led to longer tension per repetition. In this case, metabolic stress may have been prioritized over the total volume (i.e., number of repetitions), as mobility exercises might induce higher neuromuscular demand early in the set, promoting rapid fatigue and longer contraction durations. Thus, even with fewer repetitions, the cumulative duration of muscular activation (TUT) remained comparable across the groups, reinforcing the idea that training stimulus is dependent not only on repetition count, but also on contraction duration and intensity [23].

Grgic and Schoenfeld [24] highlighted that periodization of training, including planned variations in TUT, can maximize neuromuscular and metabolic adaptations. This suggests that integrating different TUT into a periodized training program may be more beneficial than maintaining a single pattern over time. In contrast, despite increasing muscle strength and power, the T + LME and PAP + LME protocols did not result in TUT alterations. This may be explained by the higher intensity and shorter duration of the PAP efforts, which are designed to improve muscle power and explosiveness, but not necessarily to prolong the TUT [25]. Li and Liang [26] also suggest that PAP, although effective for rapid strength gains, may not be ideal for improving long-term muscular endurance.

These results highlight the importance of selecting an appropriate warm-up protocol for the specific training objectives. While T + LME appears to be more effective for prolonging the TUT and consequently improving muscular endurance, PAP + LME may be more suitable for objectives involving increased power and explosive strength. Therefore, the choice of protocol should be based on individual goals and specific training requirements.

The ability to maintain exercise intensity may have a direct relationship with lactate accumulation, which is often associated with muscle fatigue [27]. However, training adaptation can improve the ability of muscles to buffer and recycle lactate, thereby prolonging the time to fatigue [27]. Blood lactate levels are important indicators of muscle stress and anaerobic activity. Although there is individual variability in lactate blood levels, this did not interfere with our results because the participants were trained and had the same level of physical conditioning, and this was not changed between one session and another, since there was a 48 h interval between sessions.

In our study, lactate levels were significantly higher after the PAP + LME protocol than after the other protocols. This suggests that PAP allows increases in muscle workload and anaerobic demand, resulting in increased lactate production [28] and may reflect the greater intensity of the warm-up, which prepares the muscles for more demanding effort. However, this high lactate production also requires careful management to avoid early fatigue and ensure that muscles can sustain the effort for longer.

Therefore, the higher lactate production observed with PAP than with the other warm-up protocols reflects the high intensity of this method. This corroborates research findings that associate higher lactate levels with high-intensity protocols [29,30], while differing from other studies that suggest that lower-intensity protocols can also induce significant elevations in lactate levels [31,32].

The results of our study are consistent with the observations of González-Alcázar et al. [29], who found higher lactate levels (4.7 mmol/L) in high-volume exercise performed with light loads (~45% 1 RM) and high speed in contrast to heavy loads (~80% 1 RM) and reduced speed (2.0 mmol/L). These findings reinforce the idea that intensity and speed of execution have a significant impact on lactate production. Similarly, MacDougall et al. [30] observed variations in the lactate peak depending on the number of sets performed at a constant load intensity (80% 1 RM), with values not exceeding ~3.5 mmol/L for one set and ~4.7 mmol/L for three sets in unilateral biceps curl exercises.

However, several studies reported conflicting results. Mavridis et al. [31] reported lactate concentrations between ~3 and 9 mmol/L for 1 min performances with 30 repetitions and loads ranging from 10% to 40% 1 RM, indicating that long-duration and lower-intensity protocols can also induce elevated lactate levels. Furthermore, Gorostiaga et al. [32] reported lactate peaks of 4.2 mmol/L during unilateral knee extension exercises with eight repetitions at 80% 1 RM, in contrast to our findings of higher lactate levels with PAP.

These discrepancies can be attributed to different training methodologies, individual variability in exercise response, and specificities of the protocols used. Despite that, training adaptation is essential for improving lactate buffering and recycling capacity, prolonging performance capacity, and delaying fatigue.

Integrating objective assessments, such as lactate levels, with subjective indicators, such as the OMNI-RES and VAS, provides a more complete view of the physiological and perceptual responses to training, allowing for precise adjustments in exercise programs to optimize performance and avoid early fatigue. The OMNI-RES is an essential tool for monitoring the intensity of perceived exertion (RPE) during strength training.

Foster [29] showed that RPE tends to be higher in protocols involving high intensity and significant anaerobic demands. Zhang et al. [28] showed that greater lactate accumulation and imposed metabolic stress after PAP may temporarily increase the perception of effort. However, other studies, such as that by Senna et al. [33], suggest that the OMNI-RES may be lower in protocols that include longer pauses between exercise sets, allowing for better recovery and less lactate accumulation. In our study, there were no significant differences in the OMNI-RES between the groups, which suggests that the three warm-up protocols did not affect the perception of exertion differently.

The difference observed between different studies may be attributed to the variability in warm-up protocols and individual characteristics of participants, such as fitness level and previous experience with the type of exercise.

The literature suggests that familiarity with the warm-up protocol and adaptation to training can significantly influence the OMNI-RES [34]. Chalchat et al. [35] indicate that, as individuals adapt to a more intense training protocol, such as PAP, the perception of exertion may decrease, reflecting an improvement in muscular endurance and exercise efficiency. However, in our study, this adaptation was not observed, possibly because of the short intervention period or individual variability of the participants. Therefore, our study highlights the importance of considering the OMNI-RES as a subjective but critical measure for adjusting and optimizing strength-training programs.

Intense warm-ups can increase the perception of pain owing to the greater production of metabolites that cause muscle fatigue [36]. However, a well-structured warm-up can help minimize this perception by better preparing muscles for subsequent effort [19]. Pain assessment can be measured using a VAS, and it is crucial for understanding the impact of training on muscle discomfort. In our study, pain levels were significantly higher after the PAP + LME protocol, reflecting that the greater intensity of the warm-up resulted in an increase in the recruitment of fast-twitch fibers and was reflected in the increase in lactate production [36]. The results of our study are consistent with the findings of Alves et al. [37], who indicated that the intensity of the warm-up is directly related to an increase in the perception of pain due to the accumulation of metabolites such as lactate. This accumulation can cause discomfort and muscle fatigue, especially in protocols that require high anaerobic demand, such as PAP.

However, other studies have reported different findings. For example, Clemente-Suárez et al. [38] suggested that warm-up protocols that include moderate-intensity exercise may not significantly increase the perception of pain owing to a lower accumulation of lactate. This differs from our findings, where PAP resulted in higher levels of pain, possibly due to the higher intensity of the warm-up. Tesarz et al. [25] highlighted that the perception of pain can vary significantly with the experience of the practitioner and familiarity with the training protocol. In our study, the variability in the response of the participants to the PAP + LME protocol may be related to these factors, suggesting that adaptation to training may decrease the perception of pain over time.

The discrepancy between the large effect size for pain perception (VAS; η^2^p = 0.35) and the null effect for perceived exertion (OMNI-RES; η^2^p = 0.00) likely reflects the distinct constructs measured by each scale. The OMNI-RES captures the perception of general physical effort during the task, which may plateau in trained individuals performing submaximal resistance sets close to failure, particularly under standardized loading conditions (75% 1 RM). In contrast, the VAS assesses local discomfort or pain, which can be more sensitive to neuromuscular fatigue and mechanical tension induced by the post-activation potentiation (PAP) condition.

Thus, although perceived exertion remained stable across the protocols, the PAP + LME session likely produced greater localized muscular stress, resulting in higher pain perception without a corresponding increase in the global effort perception [25,38]. The results suggest that while PAP may increase the perception of pain due to the high intensity of the warm-up, a well-structured warm-up protocol may help to minimize this perception and better prepare the muscles for the subsequent effort [38]. This preparation optimizes muscle efficiency and reduces early fatigue, allowing for a more consistent and effective performance during training.

In this sense, a comparison of our findings with the existing literature indicates that warm-up intensity is a critical factor for pain perception. Future studies should explore in more detail the mechanisms by which different warm-up intensities influence pain perception and muscle performance, allowing the development of warm-up protocols that optimize both performance and comfort for practitioners.

## 5. Conclusions

Our findings support the theoretical framework that well-structured, high-intensity conditioning activities can acutely improve performance not only in power-based tasks but also in strength endurance contexts.

In practical terms, PAP-based warm-ups may be useful for athletes seeking to optimize strength endurance or volume-focused training sessions. However, the associated increase in perceived discomfort should be considered when applying PAP to less experienced populations, individuals with lower pain thresholds, or rehabilitation settings. Traditional warm-up strategies may offer a more tolerable alternative when balancing performance enhancement with subjective comfort.

Future research should investigate the chronic adaptations to repeated PAP exposure, as well as the role of sex, age, training level, and warm-up configuration (e.g., load, rest interval, movement specificity) in modulating these responses. Understanding these factors may help refine warm-up prescriptions to better match individual goals and training contexts.

## Figures and Tables

**Table 1:** Descriptive characteristics of the participants (n = 12). Values are presented as mean ± standard deviation (SD).

Parameters	Mean ± SD
Age (years)	41.3 ± 5.7
Height (m) BM (kg)	1.8 ± 0.1 91.5 ± 17.0
BMI (kg/m^2^)	27.9 ± 3.1
1 RM Squat (kg)	129.3 ± 14.3

Legend: SD, standard deviation; BM, body mass; BMI, body mass index; 1 RM, one repetition maximum.

**Table 2:** Effect size comparisons related to the number of repetitions, blood lactate level, TUT, perceived exertion (OMNI-RES), and perceived pain (VAS) between resistance training sessions.

Parameters	T + LME vs. M + LME	PAP + LME vs. M + LME	PAP + LME vs. T + LME	η^2^p
Repetitions	1.25 ± 0.46 ***	3.25 ± 0.71 ***	2.00 ± 0.53 ***	0.12 (medium)
Lactate (mM)	2.23 ± 3.79	3.89 ± 3.41 **	1.66 ± 2.03	0.22 (medium)
TUT (s)	−0.97 ± 0.85 *	−1.01 ± 0.93 *	−0.05 ± 0.41	0.27 (large)
OMNI-RES (a.u.)	−0.06 ± 0.78	−0.06 ± 0.50	0.00 ± 1.04	0.00 (small)
VAS (a.u.)	1.50 ± 1.60	2.00 ± 1.58 *	0.50 ± 0.89	0.35 (large)

Data are presented as differences between measurements between training sessions and are expressed as the means ± standard deviations. The effect size is represented by η^2^p—partial eta-squared. Comparisons between the means were performed using one-way analysis of variance (ANOVA). Note: * *p* < 0.05, ** *p* < 0.01, and *** *p* < 0.001.

## Data Availability

The raw data supporting the conclusions of this article will be made available by the authors upon request.

## Abbreviations

The following abbreviations are used in this manuscript:

ST	Strength training
SS	Static stretching
DS	Dynamic stretching
PAP	Post-activation potentiation
LME	Local muscular endurance
SD	Standard deviation
BM	Body mass
BMI	Body mass index
1 RM	One repetition maximum
PAR-Q	Physical Activity Readiness Questionnaire
ICF	Informed consent form
UNIVERSO	Salgado de Oliveira University
CONEP	National Research Ethics Commission
CPET	Cardiorespiratory exercise test
PO	Power output
M + LME	Local muscular endurance during mobility
T + LME	Local muscular endurance during traditional warm-up
PAP + LME	Local muscular endurance during post-activation potentiation
VAS	Visual analog scale
OMNI-RES	OMNI Resistance Exercise Scale
TUT	Time under tension
η^2^p	Partial eta-squared
